# An Overview of Date (*Phoenix dactylifera*) Fruits as an Important Global Food Resource

**DOI:** 10.3390/foods13071024

**Published:** 2024-03-27

**Authors:** Ashgan Al-Karmadi, Anthony Ifeanyin Okoh

**Affiliations:** 1 Department of Environmental Health Sciences, College of Health Sciences, University of Sharjah, Sharjah P.O. Box 27272, United Arab Emirates; 2 SAMRC Microbial Water Quality Monitoring Centre, University of Fort Hare, Alice 5700, South Africa; aokoh@ufh.ac.za

**Keywords:** date fruit, *Phoenix dactylifera*, nutritional composition, cultivation practices, economic importance, contamination’s challenges of date fruit

## Abstract

Dates are the fruits of the date palm belonging to the *Arecaceae* family; they comprise over 2500 species and 200 genera and constitute an essential part of the daily diet worldwide, with beneficial nutritional, health, and economic values. Several varieties of date palm (*Phoenix dactylifera*) fruit exist globally, especially in hot and humid regions. This review is an overview of date palms as a significant global food resource, including their historical significance, nutritional composition, cultivation practices, economic importance, and health benefits. The historical journey of dates goes back to ancient civilizations where they were revered for their richness in essential nutrients and natural sweetness. Today, dates are a vital crop in arid regions, contributing substantially to the agricultural economy and livelihoods of communities. This paper further explores the cultivation techniques employed to enhance date production. Furthermore, the nutritional composition of dates is analyzed in detail, highlighting their high content of vitamins, minerals, dietary fibers, and antioxidants. These attributes make dates a delicious treat and a valuable nutritional component, offering numerous health benefits. The potential health effects, including improved digestion, enhanced cardiovascular health, and increased energy levels, are discussed. Additionally, this paper delves into the economic significance of the date industry and its global trade.

## 1. Introduction

In today’s increasingly sustainability-focused endeavors, the unassuming date fruit has captured the attention of researchers and food enthusiasts alike. Beyond its delightful sweetness, dates hold deep historical significance and are revered across cultures as a vital component of global food security. The date palm (*Phoenix dactylifera*) is a flowering plant cultivated principally for eating its fruit. Date trees can reach lengths of 21 to 23 m, with leaves that can attain heights of 4 to 6 m and have about 150 leaflets each [1]. The trees typically grow individually or in clusters from a single root system. Approximately 100 million date trees are cultivated worldwide, with the majority of them in the Middle East [2]. In addition, dates can be found in other countries, such as Australia, Mexico, South America, southern Africa, and the United States [3,4]. The date fruit is made up of pericarp, mesocarp, endocarp, and seed (kernel or pit) [5]. The main component is the mesocarp, which comprises parenchymatous cells divided into an outer and an inner mesocarp with layers of tanniferous cells in between [5]. The fruits undergo several developmental stages, including Khalal, Tamr, Hanabauk, Kimri, and Rutab, as they are called in the Middle East [2].

The date palm belongs to the *Arecaceae* family, a group of monocotyledonous plants classified under Angiosperms [6]. This diverse *Arecaceae* family comprises over 2500 species and 200 genera, showcasing a wide variety of palm plants with unique characteristics and features [7]. One genus, *Phoenix*, has about 14 species that are indigenous to southern Asian and African tropical or subtropical climates [8], among them is the *Phoenix dactylifera* L. The word “finger-bearing” in the species name “*Dactylifera*” refers to the clusters of fruits that this plant produces. *Dactylifera* is a combination of the Latin term *ferous*, which means “bearing”, and the Greek word *dactylus*, which means “finger” [9]. The global geographic distribution of the “*Phoenix*” genus is detailed in Table 1, as documented by Al-Alawi et al. [10]. More recently, the date palm tree genome was re-sequenced, yielding insights into the diversification of a date palm fruit crop [10]. Because date palms can thrive in harsh environments and the small fruit is stuffed with nearly all the essential vitamins, minerals, lipids, proteins, and carbohydrates, people in older times planted palm trees to use the dates fruits as food. Indeed, the Middle Eastern population consumes date fruits throughout the year, especially during the holy month of Ramadan and in special ceremonies. It is also widely used for making desserts and pastries.

## 2. Date Palm Origin

Numerous artifacts from ancient civilizations, including stone panels and coin engravings, include historical references to the date palm [11]. While this provides some helpful historical context, it has not been quite evident how the date palm has changed throughout the years. According to some historical references, the date palm was domesticated for the first time over 6000 years ago in the Persian Gulf, where it was then brought into North Africa [12]. However, the nature of its history and whose relatives contributed genetically to our modern-day date palm have not been totally understood [12].

Date culture was most likely developed as early as 3000 BCE, according to the earliest evidence from Iraq (Mesopotamia) [13]. The exact origin of the date is unknown because of its lengthy history and the extensive exchange and dissemination of date cultivars. However, it most likely originated from the ancient Mesopotamia region (Southern Iraq) or western India. Date farming began on the Arabian Peninsula and quickly extended to North Africa and the Middle East [4]. By the middle of the second millennium BCE, date culture had reached Egypt [11]. Later, as Islam spread worldwide, date farming followed and eventually reached southern Spain and Pakistan [4]. Over the past centuries, dates were also introduced to new areas such as Australia, India, Pakistan, Mexico, Southern Africa, South America, and the United States [14]. The Middle East’s history has greatly benefited from date cultivation. In the desert areas, a sizable community could not have been sustained without dates [4]. Dates have been cultivated in many nations where they are a staple food and a major source of wealth for the local population. These countries have benefited much from the dates’ enormous contributions to their economies, societies, and environments [4].

## 3. Date Palms Cultivation

Date palms can be cultivated in the early spring or fall seasons. Due to the date palm’s colossal size, ample space is required for its cultivation. The soil needs to drain well and receive a lot of sunlight. It is essential to ensure that when a young palm grows, it is not overly shaded by adjacent plants. Immature palm trees can also be cultivated in containers [15]. The date palm can withstand drought, especially after it has germinated. Nevertheless, successful palm tree cultivation requires special care and consistent hydration during its flowering and fruiting season [16].

Additionally, young trees require more water than mature trees as their soils need to be well moisturized [17]. Temperature and humidity are essential factors for cultivating date palms, and the plant requires warm, dry, and sunny weather [18]. Indeed, pollination cannot occur at temperatures below 95 degrees Fahrenheit, and though the date palm can withstand temperatures as low as 20 °F, colder temperatures are harmful to the fronds [19]. The fruit of the date palm thrives in dry weather as well, and rotting can result from excess moisture and humidity [16].

Different methods have been proposed for date palm irrigation. The oldest and most affordable method is flood irrigation, although it is labor-intensive, inefficient, and unsuitable for sandy soil [20]. The main disadvantages to the use of flood irrigation in date palm cultivation are that it uses a lot of water and is susceptible to evaporation [21]. Basin irrigation is another method used for applying water to a region that is normally leveled to a zero slope and encircled by check banks or dykes to stop runoff [22]. Land that is level and encircled by low bunds that stop water from flowing to nearby farms is called a basin. It is one of the most often used forms of surface irrigation on farms, which achieves excellent uniformity in water delivery [22], with remarkable advantages, such as low startup costs, low ongoing costs, and simple use [23]. Still, disadvantages such as labor-intensive tasks and interference with mechanical processes should be considered in this method [20]. Sprinkler irrigation is another method that works by using the spray head and extensive piping system that can cover a huge area in term of irrigation [24]. The advantages of this method is less demanding and uses water more effectively [25]. However, it has drawbacks, such as high costs and is not recommended for young palm trees since water can penetrate the heart of the palm and damage the outcome [26]. Another approach is drip irrigation, which recognized as the best option because it has low operating costs, is not affected by wind, can be automated, and requires less labor [27]. High initial costs and the need for immaculate water are drawbacks of the drip irrigation method [20].

## 4. Biology and Types of Date Palms

The monocotyledonous, perennial, and diploid (2n = 2x = 36) date palm is a member of the *Palmaceae* family [28]. The dioecious date palm has separate male and female trees. Numerous accounts of trees becoming hermaphrodites or male trees acquiring female traits have been made over the years [4]. Female and male tree inflorescences have different morphologies. Both are wrapped in a tough, fibrous covering called a spathe that shields the developing flowers from heat and sunshine [4].

The flowers are carried on a flat, tapering peduncle or rachis in female trees, also called the “fruit stalk”. The inflorescence consists of several spirally arranged, unbranched rachillae, also referred to as “strands”, on the rachis. Typically, both male and female flowers have three petals and three sepals. Female flowers are often yellowish green, whereas male blossoms are typically waxy white. The inflorescence appears in the leaf axis just before flowering, pushing through the sheaths and at anthesis, while the sheaths split longitudinally. Only the section of the rachilla that bears flowers is revealed. The fruit stalk stretches out and extends the part of the inflorescence that does not contain flowers to a length of 60 to 120 cm, fifty to sixty days after anthesis. The fruit typically grows after fertilization from one of the three carpels within each pistillate bloom. Natural fruit drop happens 25 to 35 days after the spathe cracks, and some cultivars experience a second fruit drop 100 days later [4].

## 5. Date Palm Cultivars

Globally, the date palm boasts an astonishing array of over 6000 varieties, showcasing a remarkable intra-species diversity of this fruit across different geographical regions [29]. The rich history of date palm cultivation has given rise to numerous cultivars and varieties, each distinguished by unique shapes, sizes, and organoleptic properties [9]. Morphological characteristics, encompassing features of both the trees and their fruits such as leaves, spines, and fruit shape, serve as key identifiers for date cultivars. Furthermore, the variability in color, flavor, texture, and genotypic traits further contributes to the distinctiveness of these cultivars [30]. Predominantly concentrated in the Middle East and North Africa, the majority of date cultivars flourish in these regions. Notably, the United Arab Emirates boasts around 40 million date trees, with 8.5 million thriving in the Al Ain region alone [31]. Saudi Arabia hosts approximately 31 million date trees [32], while Algeria and Libya collectively harbor nearly 30 million date trees, with 18 million in Algeria and 10 million in Libya. Despite the commercial underrepresentation of several date palm varieties, the date fruit remains a significant and marketable commodity [33]. Table 1 provides an overview of some economically noteworthy date palm cultivars from major date-producing countries, Table 2 and Table 3 present the various species of date palm fruit.

## 6. Date Palm Preservation Method

Four categories of date fruit can be distinguished and include fresh (used as fresh, Barhee variety), wet (maturation achieved by storage at low temperature or refrigeration, Hayany variety), semi-dry (Deglet Noor and Medjool varieties), and dry (Ameri, Halawi, Khadrawy, Thoory, and Zahidi varieties). The distribution, consumption, and storage of date fruits are also conducted in accordance with the amount of moisture they contain, such as the sweet Khalaal (yellow or red with 50–85% moisture content), the Rutab (light brown with 30–45% moisture content), and the Tamar (amber to dark brown with 25%) [52]. Fruits that have reached the Tamar stage are immune to microbial contamination. There are several methods to preserve the date fruits, and one important method is refrigeration, which helps to slow down the enzymatic reactions, as well as microbial and insect activities. The disadvantage of refrigeration depends on the effectiveness of low temperature directly involved after harvesting and the degree of low temperature used to preserve date fruit. Insect infestation does not occur at temperatures below 4 °C, yet cooling diminishes it; thus, even at these low temperatures, the insects may not necessarily be wiped out [53].

Fumigation is also used for date palm preservation because it kills insects in all of their developmental stages, including eggs, larva, pupa, and adult. However, the challenge in this method is that fresh fruits or those kept in deep refrigeration cannot be treated. The typical practical dose is 15 g/m^3^ for 12–24 h at temperature above 16 °C. Fumigants need time to act because it does not stop insects from leaving dates (disinfestation), and insects have acquired tolerance to this gas in a number of different places. Also, after the treatment, the residual levels remain within the MRL (maximum residual levels) [54,55]. Insects in dates can be killed by using the heating method; however, the process results in discoloration and separation of the fruit’s skin from the flesh [55,56,57,58]. The most effective method to maintain the quality of dates while having little impact on the emergence of physiological disease signs is Modified Atmosphere Packaging (MAP) [45,59,60], which is described as “packaging a perishable product in an atmosphere that has been modified so that its composition is different from that of air” [59]. The edible coating can enhance date visual appeal, safeguard fruits, and lessen stickiness, while using soft dates also maintain fruit quality by reducing water evaporation from the fruit, which can prevent weight loss by up to 50%; however, some negative impacts are focused primarily on sensory qualities and flavors [52]. The primary benefits of the UV-C light are that it leaves no residue, kills most microorganisms, is simple to use without complicated safety equipment, and is not subject to any legal restrictions. But after UV-C treatment, cells exposed to visible light undergo enzymatic photo-repair and express excision-repair genes, which may help restore DNA integrity [61].

## 7. Nutritional Value of Dates

Energy can be produced from sugar while absorbed in the body. Date fruits contain a good percentage of sucrose, fructose, and glucose varying between 65% to 80% depending on the types and maturity of the date fruit [9]. Average protein composition has been reported to be between 1.22 and 3.30% [57]. Table 4 presents the chemical makeup of methanol extracts of date palm types in a study reported recently by Assirey [62]. The study illustrated that Suqaey has the highest protein content, followed by Anabarah and Khodari, and the relative abundance of protein in the extract suggests that dates are poor in protein content [62]. However, protein levels drop during the non-enzymatic browning and tannin precipitation stage [29]. Date fruits have also been reported to contain 0.11 to 7.33% of fat [57]. In contrast, the fat content of Khodari and Suqaey was the lowest of all the date samples included in Assirey’s study [58] and ranged from 0.18 to 0.52 g/100 g. Also, the ash content ranged from 1.43 to 6.20% [57], but in another study, the ash contents of Anbarah and Suqaey were 2.33 and 2.29 g/100 g dry weight, respectively [63].

**Figure 1:**
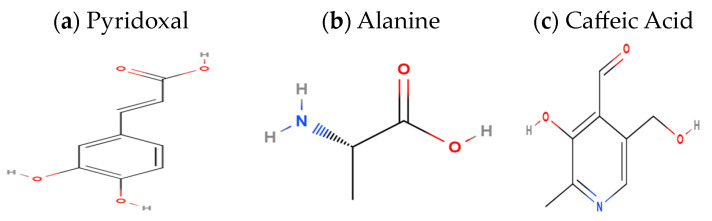
Chemical structures example for (**a**) vitamin B6, (**b**) type of amino acid, and (**c**) type of phenolic compound.

Date fruits provide the human body with a high amount of carbs, around 65.8 to 88.02% [57]. Anabarah has the highest carbohydrate content (77.3 mg/100 g), followed by Suqaey (75.3 mg/100 g) and Ajwa (72.1 mg/100 g) [63]. The dates fruit is also rich in varieties of B-complex vitamins as described the chemical structured of B6 on Figure 1a and amino acids as show in Figure 1b [58]. Dietary fiber has significant therapeutic effects, and its concentration varies depending on the type of food and its developmental stage [63]. However, dates fruit fibers ranged from 1.9 to 16.95%, of which 84 to 94% is insoluble fiber and 6 to 16% is soluble fiber [64]. Due to their low fat and cholesterol content, and sufficient fiber level, which might be beneficial to the digestive system, date palms are particularly desirable for human health [62].

The fruit of the dates palm has the potential to be a significant source of energy for humans. The amount of energy (kcal/100 g) of the date fruit cultivars: the energy values of Khodari growing in the El-Qaseem region had considerably greater (*p*  < 0.05) energy values, followed by Suqaey and Anbarah. The calorie content of the four date varieties that were chosen ranged from 265 to 287.5 kcal/100 g in Khodari and Ajwa [62]. These findings showed that palm dates from the Khodari and Ajwa varieties are potentially important for human health. They are a significant dietary source of antioxidant compounds and total phenol as in Figure 1c [65], and this suggests that, in comparison to other dried fruits, dates contain high calorie and carbohydrate contents but low percentages of protein and fat [60,62,66].

## 8. Date Palm Pollen

The word “pollen” refers to the tiny, dust-like grains found in flower anthers, which serve as both the source and transportation for male gametes [67]. Pollination is the process by which pollen grains are carried from the male anthers to the female stigma, where they germinate and fertilize the flower’s ovule, allowing for the development of seeds and fruits [11]. Traditionally, the male gametes are transported from the pollen grain to the ovule by the germination process, in which the pollen grains produce a tube-shaped extension (pollen tube) after entering the flower stigma [68]. Also, a process known as double fertilization occurs when two sperm cells released from the pollen tube unite with the egg cell and the central cell in the embryo sac, respectively, to produce a zygote and a primary endosperm [67]. The zygote finally matures into a seed embryo [69].

Dates palm pollen (DPP) is an excellent natural dietary food supplement for humans due to its nutritional value and richness in minerals, vitamins, and amino acids [70]. For example, a 100 g of DPP has an energy value of 310.88 Kcal and the protein, carbohydrates, and fat make up 40%, 19%, and 12% in total content [67]. With respect to minerals concentration, the following have been reported: potassium (750 mg/100 g), calcium (560 mg/100 g), magnesium (318.7 mg/100 g), and iron (226.5 mg/100 g), zinc (124.4 mg/100 g), and manganese (70 mg/100 g) [67]. Due to its high concentration of flavonoids and bioactive volatile unsaturated fatty acids, which play significant roles as antioxidants, anti-cancer agents, and nutritional boosters in humans, DPP can also be regarded as a functional food [71]. A recent study investigated the fortification of yogurt with DPP based on these nutritional qualities and found that it increased viscosity, syneresis, and water retention capacity [67]. In traditional or folk-lore medicine, DPP has also been used to improve fertility in both men and women and to boost libido. According to a recent study by Salmani et al. [72], consuming DPP increases testosterone and follicle-stimulating hormone levels and sperm motility in male patients. It has also been documented that DPP could improve several aspects of female sexual functions [73]. Therefore, DPP was a healing substance for rejuvenation in ancient Egyptian civilization [11]. Human fertility rates are currently trending downward, primarily in industrialized nations, and DPP has been suggested as a potential strategy for resolving this challenge [67].

## 9. Therapeutic Applications

Due to their higher exposure to sun and higher temperatures than other fruits, date palm fruits have been found to have the greatest concentration of total polyphenols among dried fruits in previous investigations [74].

Antioxidants have become of enormous interest because they scavenge free radicals linked to a variety of diseases, including cancer, heart disease, Alzheimer’s, and Parkinson’s disease [75]. When the body’s capacity to detoxify the reactive intermediates is surpassed by the rate at which reactive oxygen species (ROS) are produced, oxidative stress is a result [76]. To defend itself against free radicals, the body naturally creates antioxidants such as superoxide dismutase (SOD), catalase, and glutathione peroxidase (GSHPx) [76]. The antioxidants neutralize free radicals, making them safe for other cells. All of the free radicals produced by the body cannot be destroyed by these endogenous antioxidants [75]. Al-Alawi et al. [10] examined the cardioprotective effects of lyophilized aqueous extract of date palm fruit (Ajwa variety) in both ex vivo and in vivo settings. They observed that the extract increased cardio-myoblast cell proliferation by up to 40%, inhibited lipid peroxidation, and prevented the consumption of endogenous antioxidants [10]. Other studies investigated the therapeutic effect of *P. dactylifera* fruit extract on alloxan induced rates and reported promising antidiabetic activity of active phytoconstituents, such as flavonoids [77]. The strong nephroprotective potential against gentamicin-induced nephrotoxicity was also observed in the flesh and pit extract of *P. dactylifera* [77]. According to these investigations, nephrotoxicity caused by dichloroacetic acid in Wister rats was treated with a 4 mL/kg dose by reducing malondialdehyde (MDA) and glutathione (GSH) levels. The ability of dates to encourage and facilitate late-term labor in expectant women has also been reported [78]. These findings collectively underscore the multi-dimensional therapeutic value of date palm fruit that warrants further investigations. Table 5 summarizes the therapeutic applications identified in the literature.

## 10. Anti-Bacterial Activity of Date Palm

In developing countries, infectious diseases have a significant contribution to health issues [79]. Numerous plant species are good sources of antimicrobials, and a substantial number of plants have been shown to have physiological and therapeutical values [80]. A huge proportion of antibiotic medications in the market today come from natural or partially manufactured sources. Still, plants or herbal species have not received adequate evaluation as antibiotics despite the fact that hundreds of them have been examined for their anti-bacterial capabilities [81].

The digested date extract (DDE) and the date polyphenol extract (DPE) were evaluated for the beneficial bacterial changes that occur in the intestine while eating whole date palm fruit extract [82]. For example, the findings of Alsarayrah et al. [81] were derived using pH-controlled fecal cultures that mimicked the human large intestine. The rise in bacterial growth within 24 h was higher for those who took whole date extract (DDE) compared to those who took the extract rich in polyphenols (DPE), according to the fluorescence microscopic count, which showed an apparent increase in bifidobacteria growth in response to both extracts [81]. Due to bacterial metabolism, the flavonoids *aglycones* (*myricetin*, *luteolin*, *quercetin*, and *apigenin*) were formed in less than an hour [81]. These findings demonstrate that DDE and DPE extracts from dates can potentially suppress pathogens, significantly improve the number of beneficial bacteria, and increase the generation of lactate and acetate, all of which contribute to improving colon health. Another study examined the biochemical composition, secondary metabolites (phenolic compounds, flavonoids), and antimicrobial effect of various Emirati dates (*Phoenix dactylifera* L.) [83]. The study showed that the ethyl acetate extract of the selected cultivars inhibited *Staphylococcus aureus* and decreased the population of *Escherichia coli* [83]. These studies demonstrated that the date extract has antimicrobial potential that can be further exploited.

## 11. Contaminations in Date Palm

Fruits may naturally include chemical pollutants added during crop cultivation, post-harvesting, and other processes. Pesticides, banned additives and colors, heavy metals, stable organic pollutants, and other substances are a few of the chemical dangers and factors connected to chemical contamination in fruits [84]. Also, food additives are other chemical contamination agents found in dates. Food additives are compounds intentionally added to food for one or more technical goals but are not typically consumed as food. They include those for acidity regulation, antioxidants, colors, emulsifiers, preservatives, stabilizers, sweeteners, and thickeners. International or national authorities regulate the approval of substances for use as food additives, the identity and purity requirements of approved additives, the maximum use levels at the different commodities in which they may be used, and the food items in which their use is unacceptable [85]. Certain natural antimicrobials have demonstrated potential for usage in food items, including bacteriocins, lactoperoxidase, spices, herb leaves and oils, chitosan, and organic acids [86].

Essential oils found in spices and herbs have inherent anti-bacterial properties. Phenolic chemicals, such as ferulic oleuropein, thymol, eugenol, and the acids gallic, caffeic, and cinnamic, are the primary components of these antimicrobials. These include coriander (*Coriandrum sativum*), onion (*Allium cepa*), clove (*Eugenia aromatica*), sage (*Salvia officinalis*), rosemary (*Rosemarinus officinalis*), and garlic (*Allium sativum*). It has also been demonstrated that the oils of bay leaves, cloves, cinnamon, and thyme are very efficient against food-pathogenic microbes such as *Salmonella enteritidis*, *Escherichia coli*, *Staphylococcus aureus*, and *Listeria monocytogenes*. It is thought that compared to Gram-negative bacteria, Gram-positive bacteria were more susceptible to suppression by plant essential oils [86].

Heavy metals are widely dispersed in the environment and are released through human activity (e.g., industry, military, and agriculture) and natural activities (e.g., volcanic activity) into the air, water, and soil where plants can use them. Numerous sources release significant amounts of garbage, sewage, chemicals, and energy into the environment. Heavy metals, including cadmium, lead, and mercury, which are harmful to both people and wildlife, are present in some of these materials. Heavy metals have negative health consequences on the body, including cancer, genetic mutations, nervous system issues, and kidney failure [87].

Due to the excellent nutritional value of fruits such as dates and their widespread consumption, heavy metal contamination cannot be understated. Eating fruit containing heavy metals considerably decreases some of the body’s vital nutrients and is likely to weaken the body’s immune and defense systems. Heavy metals are harmful pollutants because they are poisonous, even in small amounts. The recurrent application of sewage sludge and industrial effluent to agricultural goods can lead to heavy metal pollution, which can have long-term consequences on soil microorganisms [88]. According to Chamon et al. [29], the heavy metal contamination of date palms exceeded the limit after collecting samples from Bangladesh and Madinah; the analysis of these samples illustrated that the concentration of Ni and Cd in some date fruits exceeded the maximum permissible limit. The acceptable daily intake in all samples of date fruit was determined to be less than the maximum permitted tolerable daily intake.

With the increase in human activity and vehicle traffic, the hazard indices of the samples taken from the new market and Badamtali overtook the unit value, indicating a possible health risk associated with consuming the date fruits that were studied. According to the study’s findings, most heavy metals under investigation were safe to eat. They were below the maximum allowable limit (MAL) range in specific date samples [89]. Also, pesticides have been implicated in dates. A compound or a combination of substances used to prevent, get rid of, or inhibit pests is referred to as a pesticide. Pesticides fall under many different categories, including fungicides, herbicides, insecticides, and many others. Pesticides are applied to preserve the quality of crops and prevent disease.

While using these chemical pesticides boosts a farmer’s output, incorrect pesticide use puts consumers, other species, and the environment at health risk [90]. A study in Egypt was conducted to test the residual limit of the pesticides in date fruit [91]. A total of 257 date samples were analyzed to check for 450 pesticide residues. They found 31 distinct pesticides in the date samples, along with the lowest, maximum, median, and percentages of the violated measured levels. Overall, the findings demonstrated that 45.92% of the samples were free of pesticide residues. In comparison, 54.09% of the samples were contaminated with pesticides, with 25.29% having higher concentrations than allowed [32].

## 12. Microbiological Contamination of Dates

The natural environment, post-harvest treatment and processing can affect the growth of microbes. Fruits and vegetables may become contaminated with pathogenic microorganisms at any time from the farm to the table because they frequently come into contact with soil, insects, animals, or people. The contamination can occur through feces, human handling, harvesting equipment, processing, transportation, and distribution [92]. Inadequate handling during loading and unloading, mixing and displaying with raw goods and animals/animal products, as well as their exposure to unhygienic surfaces and water at the point of sale, can all result in contamination and/or cross-contamination [93]. Microbial contamination of dates, especially by bacteria and fungi, has been widely reported. A typical example of such bacteria is coliforms. Coliform bacteria are rod-shaped, Gram-negative, non-spore-forming, motile or nonmotile bacteria. When cultured at 35–37 °C, these bacteria may ferment lactose and produce acid and gas. They are frequently used as a gauge for the wholesomeness of food and water [94]. Hamad [95] reported in a study in Saudi Arabia that 65% of the date samples tested contained coliforms. People who are infected by coliform may develop hepatitis, fever, diarrhea, and abdominal cramps [95]. Also, urinary tract infections may result from coliform exposure [96]. A key member of the coliform bacteria group is *Escherichia coli*; some strains of this bacteria have been known to be pathogenic.

Indeed, in the 21st century, diarrhea is still one of the leading causes of morbidity and mortality, and diarrhealic *Escherichia coli* is mainly responsible for it. Notably, traveler’s diarrhea and hemorrhagic colitis are also prevalent, particularly in developed countries. Diarrhea-associated hemolytic *E. coli (DHEC)*, enteropathogenic *E. coli* (EPEC), enterohaemorrhagic *E. coli* (EHEC), enteroinvasive *E. coli* (EIEC), enterotoxigenic *E. coli* (ETEC), enteroaggregative *E. coli* (EAggEC), and cytolethal distending toxin (CDT)-producing *E. coli* are the seven classes of diarrheagenic *E. coli* [97]. Bacterial contamination has been tested in previous research on fruits and vegetables with alarming results. For example, one recent study in Nigeria examined sixty samples of date fruits (*Phoenix dactylifera*) that were randomly selected from Kaduna’s five main marketplaces [98]. The result showed that samples of date fruit after isolation were contaminated with *Escherichia coli* (12.5%).

Another example of bacterial contamination of dates is caused by *Staphylococcus aureus*. *Staphylococcus aureus* is the second-most destructive bacterial contamination of date palm tissue culture. It is a facultatively anaerobic, gram-positive *coccal* bacterium that is one of the many prevalent skin and nasal passage flora. *Staphylococcus aureus* has been identified as a carrier in more than 20% of the human population [99]. It is one of the most common causes of food-borne illness that can contaminate food in different stages, such as preparation and processing. As the bacteria are not spore-forming, food poisoning can be avoided or reduced by heating the food. Global public health initiatives are very concerned about *staphylococcal* food poisoning, and symptoms of *staphylococcal* food poisoning appear suddenly (30 min to 8 h) and include nausea, intense vomiting, abdominal cramping, and diarrhea. Luckily, *Staphylococcal* food poisoning usually resolves within one to two days after onset and is self-limiting. However, it can result in life-threatening infections in children, older adults, and those with impaired immune systems [100]. A study in Nigeria reported high counts of bacteria in hard and soft date fruits in the order of 10^5^ CFU/mL [61], which is way above the 10^4^ CFU/mL recommended for food for human consumption [101].

Fungi have also been implicated in date contamination. Four elements are required for mold to thrive: water, food, suitable air quality, and temperature. Furthermore, mold can grow on food that includes any amount of water or fluid, and it can only develop if it has available food to grow. Mold is a fungus that feeds on rotting or dead organic materials, which can harm health and food quality. Moreover, mold tends to thrive in dark, damp, and chilly surroundings, yet it can also be found in warmer climates [102]. Dates can be contaminated with mold during storage and transport; hence, low temperatures are recommended for the storage and transportation of dates to maintain their quality and prevent the growth of molds.

Depending on the cultivar, the ideal temperature for Tamar date preservation is 0 °C for 6 to 12 months. However, semi-soft dates like “Deglet Noor” and “Halawy” have an extended preservation life compared to soft dates like “Medjool” and “Barhee”. For more extended preservation periods, temperatures below −15.7 °C are recommended [57]. Finally, dates with 20% moisture or less can be stored at −18 °C for longer than a year, at 0 °C for a year, at 4 °C for eight months, or at 20 °C for one month at humidity between 65 and 75% [57]. In a study [103], all date fruit cultivars were contaminated with fungus, with the most prevalent species being *Curvularia lunata* (15%), *Fusarium monoliforme* (19.83), *Aspergillus terreus* (30%), and *Rhizopus stolonifer* (38.33%), *Fusarium solani* (34%), *Fusarium oxysporum* (43.83), *Aspergillus flavus* (46.83%), *Penicillium stolonifer* (55.16%), *Alternaria alternate* (55.66%), and *Aspergillus niger* (81%). Aflatoxins, which are mycotoxins, are known to cause diseases like cancer and harm vital organs, including the liver, kidney, brain, and nervous system [103].

The devastating impact of mycotoxins on the immune system of both adults and children is of major public health concern, while newborns are more vulnerable to mycotoxins than adults due to their smaller body weight and less acidic stomach [104]. Abass et al. (2013) examined the most common species of fungi in six different date palm cultivars in Iraq, including Um Al-Dihin, Shwaythee, Breem, Barhi, Hilawi, and Al-Sayer. They documented fungal contamination with *Aspergillus niger*, *Aspergillus clavatus*, and *Alternaria alternata*, with *Aspergillius niger* and *Alternaria alternata* being the most prevalent fungi in infected date palm tissues [99]. In another study in Morocco, the microbial qualities of several of the date samples did not meet the permitted limits [105]. In South Algeria, thirty samples of the hmira cultivar were investigated, and fecal coliforms, *Yersinia enterocolitica*, and three species of *Klebsiella* (*K*. *Terrigena*, *K. pneumonia*, and *K. oxytoca)* were detected. The study also observed a sizable amount of fungi microflora with counts ranging from 2.36 log to 5.18 log cfu/g comprised mainly of *Saccharomyces cerevisae*, *Zygosaccharomyces fermentati*, *Hansenula anomala*, *Lodderomyces elongisporus*, *Kluyveromyces fragilis*, *Penicillium notatum*, *Rhizopus oryzae*, and *Aspergillus niger* [106].

Numerous methods from various cultures have been explored to prevent or mitigate bacterial and fungal contamination in date palms. Several antibiotics and fungicides have been introduced into the medium to control microbial contamination. When employed as prophylactics in a medium of date palm tissue culture, most chemical agents successfully prevent bacterial and fungal growth [99]. It is recommended that antibiotics and fungicides to be used should be soluble, stable, acceptable for combination therapy, affordable, non-resistance-inducing, and produce strong anti-bacterial activity and not be damaging to human health [99].

## 13. Challenges Associated with Dates Palm

Due to challenges such as water shortage, salinity of the soil and water, and low soil fertility, among others, date palm farming is difficult in areas with such characteristics. For instance, the UAE has a hyper-arid environment where reference evapotranspiration surpasses 2000 mm, although yearly precipitation is only 50 mm on average. In contrast to such abiotic pressures, insects, and diseases, particularly the red palm weevil (*Rhynchophorus ferrugineus*), also limit the output of date palms. The Food and Agriculture Organization of the United Nations estimates that pests and illnesses cause yearly reductions in global date palm plantations by 30% [22]. This illness can infect the date palm worldwide. The term of the illness varies depending on the type of infection and the morphological symptoms that have manifested. These include Theilavopsis bud rot, stem bending, dry basal rot, crazy disease (Medjnoon), inflorescence blight, terminal bud rot, trunk rot, leaf black scorch, and heart rot caused by the fungus *Thielaviopsis paradoxa*. Most age groups of palms often exhibit four unique symptoms: bud rot, heart or trunk rot, black scorch on the leaves, and inflorescence blight. Any infection may result in partial to total necrosis of the tissues. The petioles, fruit strands, and fruit stalks develop a burned, charcoal-like look due to typical lesions that are hard, dark brown to black in color, carbonaceous, and present in masses [24].

## 14. Industrial and Gastronomic Use of Dates

Because of the sugar they contain, dates are a popular fruit all throughout the world. In 2020, the world produced roughly 9.5 million tons of dates [107]. Dried date fruit is usually eaten whole. Nevertheless, certain inferior dates are also processed into various value-added goods used in the culinary industry, like date syrup, date confectionary, date candies, and date sweets. A significant amount of date seeds or kernels are produced from this procedure in addition to their direct consumption; however, on average, 10–15% of the total date mass is lost [108]. Date seed powder, which is made from dried and roasted seeds, can also be used to make a beverage that tastes and smells like coffee but without the caffeine that can lead to health problems like insomnia and hypertension. This beverage is made with similar aromatic compounds, such as alcohols and aldehydes, found in Arabica coffee [109]. Moreover, hot water is used to extract date syrup from the fruits in the date processing industry, which includes the manufacture of date syrup. The seeds and the fibrous fruit pulp material, sometimes referred to as date fruit pomace or date cake, are the two types of industrial waste that are produced as a result of this extraction procedure [110]. These lignocellulosic materials are abundant and have the potential to provide bioenergy [111], but they are usually burnt in fields or disposed of in landfills, which has negative environmental effects [112]. Date palm trees possess the capacity to generate substantial quantities of waste biomass, making them a highly promising and economical resource that can be effectively employed in biorefinery operations. With the least amount of waste production and chemical consumption, the biorefinery idea offers a viable substitute for traditional industrial processes in the conversion of biomass feedstock into a variety of renewable bioproducts and bioenergy. Because multi-product biorefineries enable the manufacture of numerous value-added products from a single feedstock, they additionally improve the sustainability of the bioconversion process [113,114].

## 15. Gap of Knowledge and Future Research Directions

A notable gap in the literature on dates fruit is the lack of detailed exploration into the potential health benefits and medicinal properties of dates beyond their nutritional values. Future research endeavors could focus on elucidating the specific bioactive compounds present in dates and their potential therapeutic applications, such as their effects on chronic diseases like diabetes, cardiovascular disorders, and cancer. Moreover, there is a need for studies examining the impact of date consumption on gut microbiota and overall digestive health. Additionally, investigating sustainable cultivation practices and post-harvest processing techniques to minimize environmental impact and maximize yield could further enhance the role of dates as a sustainable global food resource. Addressing these gaps in the literature will contribute to a more comprehensive understanding of the multifaceted benefits and potential applications of dates in promoting human health and sustainable food systems.

While there is an extensive body of literature on date palms, there appears to be scanty information on the spoilage of date palms and fruits. As well, the factors influencing the spoilage of palms and its fruit in different environmental matrices and regions require further investigations to inform manufacturing policies and practices. Identifying the agents and factors that negatively impact date farming, fruit processing, and shelving lives of the different fruit varieties require further attention, and the same holds for the valorization of the date palm and fruit wastes for value-added products. Also, while some pathogens have been reported in date fruits, their implications on humans and ecological health through relevant health risk assessments seem largely lacking.

## 16. Conclusions

The date palm (*Phoenix dactylifera*) is a resilient and versatile plant, particularly suited for hot weather conditions. Its adaptability to various irrigation methods enhances its agricultural viability. Moreover, the plants’ diverse varieties offer valuable options for cultivation, catering to different preferences and purposes. Beyond its economic significance, the date palm boasts exceptional nutritional value, serving as a rich source of essential nutrients. The plants’ utility extends far beyond their fruits; dates, palm pollen, leaves, and fibers find applications in the healthcare and agriculture industries. The date palm’s fruits, which exist as several varieties, have proven to be of rich nutritional, therapeutic, and economic values globally, especially in the Middle-East and North Africa. However, the challenges associated with date palm cultivation include potential contamination from chemicals such as pesticides and heavy metals, additives, and microbiological contamination from different bacteria. Addressing these challenges is imperative for sustaining the plants’ productivity and ensuring the safety of its products. As we move forward, suggested future research directions and sustainable practices will be vital to maximizing the benefits of the date palm and mitigating associated risks.

## Figures and Tables

**Table 1:** Species of the genus *Phoenix*, together with information about their habitats and geographical spread and phylogenetic relationship.

Species	Local Name	Geographical Distribution	Phylogenetic Relationship
*Phoenix acaulis*	Stemless date palm	Bhutan, Nepal, northern India.	F P02acaF M Q10acaM
*Phoenix andamanensis*	Andaman Island date palm	Myanmar.	F Q01andF M Q03andM
*Phoenix atlantica*	Cape Verde Island	Cape Verde Islands.	F Q22atlF M Q23atlM
*Phoenix caespitosa*	Date palm	Djibouti, Oman, Saudi Arabia, Somalia, Yemen.	F Q07caeF M Q09caeM
*Phoenix canariensis*	Canary Island date palm	Australia, Bermuda, Canary Islands, Italy, Spain.	F P08canF M P09canM
*Phoenix dactylifera* L.	Date palm	Arabian Peninsula, Australia, California, China, El Salvador, Fiji, Iran, India, Mauritius, northern and western Africa, Pakistan, Spain.	F dnPdF F khlsF2016 M dnPdM
*Phoenix loureiroi*	Mountain date palm	China, Himalayas, India, Indochina, Philippines.	F P14hanF M P13hanM
*Phoenix paludosa*	Mountain date palm	Andaman, India, Indochina, Sumatra.	F P17palF M P19palM
*Phoenix pusilla*	Ceylon date palm	India, Sri Lanka.	F Q14pusF
*Phoenix reclinata*	Senegal date palm	Africa, Arabian Peninsula, Comoros, Madagascar.	F P21recF M P20recM
*Phoenix roebelenii*	Pygmy date palm	China (Yunnan) to North Indo-China.	F P03roeF M P5roeM
*Phoenix rupicola*	Cliff date palm	Andaman Islands, Bhutan, India.	F P0XrupF M Q15rupM
*Phoenix sylvestris*	Indian date palm	Indian Subcontinent, Myanma, southern China.	F P23sylF M P25sylM
*Phoenix theophrasti*	Cretan date palm	Greek Islands, Turkey.	F Q17theF M Q19theM

F. for female and M. for male.

**Table 2:** Selection of prominent and economically important cultivars of date palm from main date producing countries.

Name	Country	Fruit Description	References
Almehtari	Iran	Yellow-light brown, soft date fruit	Karizaki [34]
Amhat	Egypt	Soft date fruit	Dorria et al. [35]
Amry	Egypt	Semi-dry date fruit	Elshibli et al. [36] Marondedze et al. [37]
Anbara	Saudi Arabia	Maroon-red, large, wrinkled date fruit	Aleid et al. [38] Zhang et al. [39]
Ajwa	Saudi Arabia	Oval shaped, black, wrinkled date fruit	Zhang et al. [39] Khalid et al. [40]
Aseel	Pakistan	Semi-dry, sweet date fruit	Markhand et al. [41]
Barhi	United Arab Emirates, Bahrain, India, Iran, Iraq, Saudi Arabia, Syria, Sudan	Red-brown, soft date fruit	Świąder et al. [42]
Bint-Eisha	Egypt	Red, soft date fruit	Elshibli et al. [36]
Dayri	Iran	Dark brown, large, dry date fruit	Karizaki [34]
Deglet Noor	Tunisia, Algeria, Libya, Saudi Arabia, Syria	Unique taste and shape (often oblong-ovate or elliptical), light brown, semi-dry date fruit	Mrabet et al. [43] Zhang et al. [39] Świąder et al. [42] Racchi et al. [44]
Gantar	Iran	Brown-red, elliptical, soft date fruit	Karizaki [34]
Gargoda	Egypt, Sudan	Dry date fruit	Elshibli and Korpelainen [36]
Hallawi	United Arab Emirates, Saudi Arabia	Medium, long, soft date fruit	UAEU [31] Zhang et al. [39]
Hayany	Egypt	Black, shiny, and oblong soft Date fruit	Omar and El-Ashry [45] Świąder et al. [42]
Hamria	Libya	Dark brown or black soft date fruit	Racchi et al. [44]
Khalas	United Arab Emirates, Kuwait, Oman, Saudi Arabia, Syria	Red-brown, elliptical to ovate, soft date fruit	Świąder et al. [42]
Khodry	Saudi Arabia, Libya	Sweet date fruit	Habib and Ibrahim [46] Racchi et al. [44]
Lulu	United Arab Emirates	Dark amber colored, oblong, soft date fruit	Świąder et al. [42]
Mabroom	Saudi Arabia	Brown, medium to large, date fruit	Zhang et al. [39]
Medjool	Morocco, India, Israel, Kuwait, Palestine, Saudi Arabia, Syria	Light to dark brown, soft date fruit	Świąder et al. [42]
Mozafati	Iran, Pakistan	Dark brown to black, cylindrical, soft date fruit	Świąder et al. [42]
Piarom	Iran	Dark brown to black, large, thin, sem-dry date fruit	Karizaki [34]
Rabbi	Iraq	Dark brown or red, long, thin, fleshy semi-dry date fruit	Karizaki [34]
Samany	Egypt	Soft date fruit	Rabie et al. [47]
Sayer	Iran, Iraq	Brown to red, rectangular ellipse shaped, semi-dry date fruit	Karizaki [34]
Shishi	United Arab Emirates, Saudi Arabia, Qatar	Dry sweet date fruit	Kamal et al. [48]
Sokkery	United Arab Emirates, Libya	Soft, sweet date fruit	Habib et al. [49]
Zaghloul	Egypt, India, Syria	Soft date fruit	Rabie et al. [47]

**Table 3:** The different species of date palm fruit as cited in Hazzouri et al. [50] and Flowers et al. [51].

Sample	Species Classification	Origin	Tissue
Kamla, Khalte, Bousl Khine, Raslatmar, Jihl, Boufkouss Rarass, Aziza, Fagous, Biddajaj,	dactylifera	Morocco	fruit
Medjool	dactylifera	Morocco	leaf
Thory, Rhars, Deglet Noor	dactylifera	Algeria	leaf
Alig, Besser Haloo, Horra	dactylifera	Tunisia	leaf
Abel, Tagiat, Hamria, Barmel,	dactylifera	Libya	fruit
Hayany, Samany, Saidi, Zagloul	dactylifera	Egypt	leaf
Jao	dactylifera	sudan	fruit
Chichi, Hilali, Rothan, Shagri, Khenezi, Nebeit Seif, Ajwa	dactylifera	KSA	leaf
Dibbas, Helwa, Hiri, Fard #4, Lulu, Abouman,	dactylifera	UAE	leaf
Nagal	dactylifera	UAE	fruit
Maktoumi, Khadrawy, Khastawi, Sultana, Um al hamam, Um al blaliz, Ewent ayob, Azraq azraq, Ebrahimi, Dajwani, Silani, Khisab, Halawy, Zahidi, Amir Haj, Manjouma, Braim	dactylifera	Iraq	leaf
Kabkab	dactylifera	Iran	leaf
Mazafati, Piavom, Rabee	dactylifera	Iran	fruit
Kashoowari, Dedhi, Naquel Khuh, Aseel, Kuproo, Began, Faslee, Karbali, Gajar, Hawawiri, Otaquin	dactylifera	Pakistan	leaf
Canariensis [DP6A]	canariensis	Gran Canaria	leaf
Atlantica [CAP1 POPMAL1]	atlantica	Maio I.	leaf
Atlantica [CAP50 BOA1]	atlantica	Boa Vista I	leaf
Reclinata [DP18]	reclinata	Rwanda	leaf
Theophrasti [THE83 91051]	theophrasti	Crete, Greece	leaf
Theophrasti [GOLK001 91020]	theophrasti	Golkoy, Turkey	leaf
Theophrasti [02a], Theophrasti [05a]	theophrasti	Epidaurus	leaf
Theophrasti [A1]	theophrasti	White Lake	leaf
Theophrasti [A5]	theophrasti	Chrisoskalitissa	leaf
Theophrasti [B1], Theophrasti [B3], Theophrasti [B5]	theophrasti	Preveli	leaf
Theophrasti [C1], Theophrasti [C4]	theophrasti	Maridaki	leaf
Theophrasti [D1],Theophrasti [D3],Theophrasti [D5]	theophrasti	Vai	leaf
Theophrasti [E1],Theophrasti [E2]	theophrasti	Almyros	leaf
Theophrasti [F1],Theophrasti [F2]	theophrasti	Drapano	leaf

**Table 4:** The chemical composition (g/100 g dry weight) of methanol extracts of four date flesh varieties as cited in Assirey [62].

Cultivars	Dry Matter	Moisture	Total Protein	Total Fat	Total Carbohydrate	Ash
Ajwa	92.00 ± 0.42	22.8 ± 0.1	2.91 ± 0.07	0.48 ± 0.03	72.1 ± 0.35	3.43 ± 0.04
Anabarah	93.64 ± 0.05	29.5 ± 0.2	3.49 ± 0.15	0.52 ± 0.03	77.3 ± 0.34	2.33 ± 0.05
Khodari	90.14 ± 0.33	19.5 ± 0.1	3.42 ± 0.12	0.18 ± 0.08	71.5 ± 0.27	3.42 ± 0.04
Suqaey	94.02 ± 0.02	14.5 ± 0.1	3.73 ± 0.10	0.41 ± 0.03	75.3 ± 0.45	2.29 ± 0.02

**Table 5:** Summary of the therapeutic applications of date palm fruit.

Applications	Health Benefit
Antioxidants	Cancer, heart disease, Alzheimer′s, and Parkinson′s disease, neutralize free radicals [75].
lyophilized aqueous	Cardioprotective effects, increased cardio-myoblast cell, inhibited lipid peroxidation, prevented the consumption of endogenous antioxidants [10].
Phytoconstituents	Antidiabetic activity [77].

## Data Availability

No new data were created or analyzed in this study. Data sharing is not applicable to this article.
